# Topological Organization of Center‐of‐Pressure Dynamics During Virtual Reality Walking Differs Between Individuals With Parkinson's Disease and Healthy Older Adults

**DOI:** 10.1002/hsr2.73286

**Published:** 2026-09-23

**Authors:** Elaheh Azadian, Mahdi Majlesi, Alireza Ardalany Moghadam, Makwan Jabar Ali, Rezvan Bakhtiarian

**Affiliations:** ^1^ Department of Motor Behavior Ha.C., Islamic Azad University Hamedan Iran; ^2^ Department of Sport Biomechanics Ha.C., Islamic Azad University Hamedan Iran; ^3^ Department of Corrective Exercise and Sport injury, Faculty of Physical Education and Sport Sciences Allameh Tabataba'i University Tehran Iran; ^4^ Physical Education and Sport Sciences Department University of Halabja Halabja Kurdistan Region Iraq

**Keywords:** center of pressure, entropy, gait, Parkinson disease, virtual reality

## Abstract

**Background:**

Gait impairment in Parkinson's disease (PD) is multifactorial and may become more pronounced under perceptual–cognitive challenge. Virtual reality (VR) provides an altered locomotor context, yet its effects on both conventional magnitude‐based Center of pressure (CoP) metrics and higher‐order structural organization remain unclear. This study compared linear, nonlinear, and topological characteristics of stance‐phase CoP trajectories between individuals with PD and healthy older adults during normal and VR walking, with particular emphasis on whether VR elicited group‐specific differences in topological organization.

**Methods:**

Fifteen individuals with idiopathic PD and 17 age‐matched controls performed overground walking under normal gait (NG) and VR conditions. Stance‐phase CoP trajectories were analyzed using linear magnitude measures (path length, velocity, root mean square, ellipse area), sample entropy (SampEn), and persistent homology–derived persistence entropy (H0 and H1). Mixed‐factor repeated‐measures ANOVA models assessed Group and Task effects.

**Results:**

VR increased CoP path length (*p* = 0.005) and SampEn (*p* = 0.015) across groups, while H0 persistence entropy decreased (*p* = 0.002). H1 persistence entropy showed a significant Group × Task interaction (*p* = 0.025), with a between‐group difference during VR but not normal walking. No Group × Task interactions were found for conventional CoP measures. Stance duration was longer in PD (*p* = 0.014).

**Conclusions:**

The primary analysis showed a group‐dependent H1 persistence‐entropy response across normal and VR walking. However, because zero entropy represented both zero‐ and one‐feature diagrams and robustness across alternative point‐cloud densities and persistence thresholds was not examined, this finding should be considered exploratory. Conventional CoP measures did not show diagnosis‐specific responses to VR. H1 persistence entropy may therefore provide complementary information about CoP trajectory organization, although its physiological and clinical significance requires further investigation.

## 1 Introduction

Gait impairment is one of the most disabling features of Parkinson's disease (PD), contributing substantially to reduced independence, increased fall risk, and diminished quality of life [1]. Contemporary perspectives emphasize that gait dysfunction in PD is multifactorial. Beyond bradykinesia and rigidity, complex interactions among cognitive processes, sensory integration, and non‐dopaminergic neural networks contribute to instability and gait disturbances, particularly under conditions that impose greater environmental or task demands [2]. Challenging contexts (e.g., dual‐tasking, turning, obstacle negotiation, or visually complex environments) often exacerbate these deficits, rendering “stress tests” of gait control valuable for identifying clinically meaningful impairments that may not be apparent during unconstrained, straight‐line walking [3].

Virtual reality (VR) provides a controlled means of altering the visual and environmental context of walking while preserving selected features of real‐world navigation [1]. In PD, immersive or task‐relevant VR environments can alter spatiotemporal gait parameters and may elicit freezing‐related disturbances when salient environmental triggers (e.g., doorways) are simulated [2]. Recent studies further indicate that VR exposure can modify gait characteristics and the expression of freezing of gait, underscoring that VR is not a neutral display modality but rather a perturbation to locomotor control [3]. In parallel, VR‐based rehabilitation has demonstrated improvements in balance outcomes in PD, as reported in systematic reviews and meta‐analyzes, supporting growing clinical interest in VR as both an assessment and intervention tool [4]. Nevertheless, the effects of VR on underfoot loading and the organization of stance‐phase CoP trajectories in individuals with PD remain insufficiently characterized.

Immersive VR may modify postural control by altering optic flow and the visual representation of self‐motion, potentially creating a mismatch between visual information and vestibular or proprioceptive cues. Processing this altered sensory context also requires attentional resources that would otherwise be available for automatic gait regulation. During stance, sensory reweighting and increased attentional demand may modify the timing, direction, and distribution of corrective forces applied beneath the foot, thereby changing the two‐dimensional geometry of the CoP trajectory. Such changes may affect not only conventional measures of CoP magnitude but also the spatial arrangement of trajectory points across multiple scales. In persistent homology, nearby points in the CoP trajectory are progressively connected as the distance threshold increases. H0 describes how initially separate connected components merge, whereas H1 identifies loop‐like regions enclosed by the connected points. Features that remain present across a wider range of distance thresholds are considered more persistent. Persistence entropy summarizes how the persistence of these features is distributed within the point cloud. Accordingly, the topological response to the altered VR context may differ between individuals with PD and healthy older adults [5]. Nevertheless, these topological features should be regarded as geometric descriptors of the CoP trajectory rather than direct measures of neural control mechanisms. These mechanisms are theoretically plausible but were not directly measured or systematically manipulated in the present study.

During walking, the CoP represents the instantaneous point of application of the resultant ground reaction force, and its trajectory describes the progression of this point beneath the supporting foot throughout stance. CoP trajectories therefore provide a dynamic kinetic description of foot–ground interaction, loading progression, and aspects of balance regulation during gait [6, 7]. However, CoP does not directly measure neuromuscular control strategies, and changes in its trajectory should not be attributed to specific neural or motor‐control mechanisms without complementary evidence. In PD, evidence suggests that dynamic CoP characteristics during walking may be altered and can exhibit clinically meaningful asymmetries [8]. Conventional magnitude‐based CoP metrics, including path length, velocity, and RMS, provide clinically and biomechanically meaningful summaries of the extent and rate of CoP displacement. However, these measures do not explicitly characterize the temporal regularity or multiscale geometric organization of the trajectory. Nonlinear and topological analyzes may therefore provide complementary descriptions of CoP dynamics without directly quantifying the flexibility, exploration, or rigidity of the underlying control system.

Among these complementary approaches, entropy‐based metrics such as sample entropy (SampEn) characterize the regularity and predictability of a time series and have been used to differentiate walking conditions and populations. However, interpreting these measures as indicators of “complexity” requires methodological caution and careful contextualization [9]. In gait research, SampEn has demonstrated sensitivity to dual‐task perturbations and to preprocessing decisions when applied to whole‐gait signals, including CoP displacement trajectories [10]. In parallel, topological data analysis (TDA), particularly through persistent homology, provides a principled framework for quantifying multi‐scale geometric and connectivity properties of data (e.g., connected components and loops) in a manner that is theoretically robust to noise [11, 12]. Persistent entropy condenses persistence barcodes or diagrams into a scalar summary measure and exhibits formal stability under defined conditions, supporting its potential as a compact descriptor of higher‐order structure [12, 13]. Previous studies have applied persistent homology to gait signals and PD classification, primarily using wearable‐sensor signals, movement trajectories, or reconstructed phase‐space representations [13, 14, 15]. However, to our knowledge, persistent homology has not previously been applied directly to reference‐translated, two‐dimensional stance‐phase CoP trajectories to compare normal and VR walking in individuals with PD and healthy older adults. It therefore remains unknown whether VR‐related changes in H0 and H1 persistence entropy differ between these populations or provide information complementary to conventional CoP measures.

The present study examined whether walking in a VR environment modifies stance‐phase CoP dynamics differently in individuals with PD and healthy older adults. To provide complementary descriptions of CoP behavior, we examined (i) conventional magnitude‐based measures, (ii) SampEn, and (iii) H0 and H1 persistence entropy derived from the two‐dimensional CoP trajectory. We hypothesized that [16]: individuals with PD and healthy older adults would differ in one or more CoP measures, indicating a main effect of Group [17]; VR walking would alter CoP measures relative to normal walking, indicating a main effect of Task; and [18] the magnitude or direction of VR‐related changes would differ between groups, indicating a Group × Task interaction. Because previous evidence was insufficient to predict the direction of changes in H0 and H1 persistence entropy, the hypotheses concerning the topological outcomes were non‐directional. This multilevel approach was intended to determine whether temporal regularity and trajectory topology provide information complementary to conventional magnitude‐based CoP descriptors during challenging walking conditions.

## 2 Methods

### 2.1 Participants

A cross‐sectional study with a mixed‐factor design was conducted to compare individuals with Parkinson's disease (PD) and age‐matched healthy older adults under two locomotor conditions: conventional overground walking (normal gait, NG) and walking performed within a VR environment. CoP trajectories were decomposed into anteroposterior (AP) and mediolateral (ML) components for analysis during the stance phase of gait. An a priori power analysis was undertaken using G*Power to determine the required sample size for a repeated‐measures ANOVA framework (effect size = 0.40, α = 0.05, statistical power = 0.80). This calculation indicated a minimum of 12 participants. A total of 32 individuals were ultimately recruited, including 15 participants with idiopathic PD and 17 neurologically intact older adults who served as the control group (CG). The diagnosis of PD was established by a qualified neurologist. Recruitment took place in Hamadan Province, Iran, through community advertisement, outpatient neurology clinics, and Besat Hospital. For the PD group, eligibility criteria included a clinical diagnosis of idiopathic PD classified as Hoehn and Yahr stage II–III (mean stage 2.5 ± 0.5) [19], assessment during the ON‐medication state, the ability to ambulate independently without assistive devices, absence of recent lower‐limb surgery, no implanted medical devices, and preserved visual and auditory function. Control participants were required to have no history of neurological or significant musculoskeletal disorders, no prior relevant surgical procedures, intact sensory function, and independent walking ability. All participants were between 50 and 70 years of age. Exclusion criteria comprised a diagnosis of atypical or secondary parkinsonism, marked motor or sensory deficits that could compromise task execution, use of psychoactive medications with potential effects on gait or balance, reliance on walking aids, or screening results indicating clinically significant cognitive impairment or substantially reduced quality of life. The study protocol was approved by the Ethics Committee of Islamic Azad University, Hamedan Branch (approval code: IR.IAU.H.REC.1401.001). All procedures were conducted in accordance with the Declaration of Helsinki, and written informed consent was obtained from each participant prior to enrollment.

### 2.2 Instruments and Examination

Three‐dimensional lower‐extremity kinematics were acquired using a six‐camera Vicon motion capture system (Oxford Metrics, Oxford, UK) operating at a sampling frequency of 100 Hz. Participants completed barefoot overground walking trials with 16 retroreflective markers positioned according to the Plug‐in Gait lower‐body model. Ground reaction forces (GRFs) were recorded synchronously using two Kistler force platforms (Type 9281, Kistler Instrument AG, Winterthur, Switzerland) at 1000 Hz. GRF signals were processed with a fourth‐order low‐pass Butterworth filter using a cut‐off frequency of 20 Hz [20].

Data were collected during overground walking along a 12‐m walkway under two experimental conditions: (i) normal gait (NG) and (ii) virtual reality gait (VRG). The order of the two conditions was randomized for each participant to minimize systematic learning, adaptation, and fatigue effects. Before formal recording, participants completed familiarization trials to become accustomed to the walkway, force‐platform locations, and, for the VRG condition, the immersive virtual environment. For the VRG condition, a three‐dimensional digital replica of the laboratory was developed using Unity (Unity Technologies, USA) and presented through an Oculus Quest 2 head‐mounted display (Meta Platforms Inc., USA). The headset provided a refresh rate of 90 Hz and an approximate field of view of 110°. Head position and orientation were tracked using the headset's built‐in six‐degrees‐of‐freedom inside‐out tracking system [21]. To establish spatial correspondence between the physical and virtual environments, the dimensions of the laboratory walkway were reproduced in Unity based on direct measurements of the experimental space. The force‐platform locations and the start and end points of the walking path were represented at their corresponding positions within the virtual environment [22]. Participants walked along the same physical walkway in both conditions, while the virtual scene provided a spatially matched representation of the laboratory layout. Visual translation and optic flow were generated directly from the participant's real‐time HMD position through the headset's six‐degrees‐of‐freedom tracking. Virtual‐camera translation was spatially coupled 1:1 to the participant's actual overground displacement; therefore, virtual forward movement was determined by the participant's self‐selected walking speed rather than by a prerecorded animation, joystick input, or independently specified virtual speed. In each condition, participants completed six walking trials at a self‐selected comfortable speed. Trial validity was evaluated using predefined criteria, including complete marker trajectories, full and valid force‐platform contact without deliberate targeting, uninterrupted headset tracking during VRG, and the absence of technical artefacts in the force signals. Trials not meeting these criteria were excluded. Three valid trials satisfying all signal‐quality criteria were retained for each participant and condition and averaged for subsequent analysis.

Center‐of‐pressure (CoP) trajectories were extracted from the force platforms for the stance phase of gait. CoP coordinates were exported from Vicon Nexus in millimeters along the laboratory mediolateral (COP_X_) and anteroposterior (COP_Y_) axes. Ref_X_ and Ref_Y_ represented the fixed coordinates of the force‐platform reference point and remained constant within each trial. Reference‐translated coordinates were calculated as follows:

$$\mathrm{Co}P_{\left\{\mathrm{ML}\right\}}^{\left\{\mathrm{rel}\right\}\left(t\right)}=\mathrm{CO}P_{X\left(t\right)}-\text{Re}f_{X}.$$

$$\mathrm{Co}P_{\left\{\mathrm{AP}\right\}}^{\left\{\mathrm{rel}\right\}\left(t\right)}=\mathrm{CO}P_{Y\left(t\right)}-\text{Re}f_{Y}.$$

This transformation shifted the coordinate origin but did not rotate the data into a foot‐aligned coordinate system or remove between‐trial differences in foot placement relative to the force platform. It therefore should not be interpreted as isolating within‐stance CoP dynamics from foot‐placement variability. Because subtraction of a fixed reference applies the same translation to all trajectory points within a trial, it does not change pairwise Euclidean distances and consequently does not affect the persistent‐homology calculations.

The resulting trajectories were low‐pass filtered using a fourth‐order Butterworth filter with a 20‐Hz cut‐off frequency. To standardize trajectories with different stance durations and ensure an equal number of observations in each two‐dimensional CoP point cloud, each trajectory was linearly resampled to 200 equidistant points. Fixed‐length normalization has previously been used for CoP and gait‐related biomechanical signals to facilitate comparisons across trials and participants [23, 24]. The selected resolution represented approximately 0.5% increments of the stance phase and provided a consistent point‐cloud density across trials. Following resampling, the ML and AP trajectories were mean‐centered so that RMS reflected variability around the within‐trial mean. Mean‐centering removed only the constant spatial offset represented by the trial‐specific mean position; it did not rotate the trajectories into a foot‐aligned coordinate system or eliminate placement‐related differences in trajectory orientation or geometry. Mean‐centering did not affect SampEn, ellipse area, or persistent‐homology measures because these metrics are invariant to constant translation.

Feature extraction was conducted at the trial level, and values were subsequently averaged within participant, condition, limb, and—where applicable—direction to derive representative metrics for inferential analysis. Four primary linear features were examined: CoP path length, mean CoP velocity, root mean square (RMS) amplitude, and ellipse area. Path length was calculated as the cumulative Euclidean distance traversed by the CoP during stance, with AP and ML components quantified as directional path lengths [25]. Mean CoP velocity was defined as path length divided by stance duration (direction‐specific). RMS amplitude was computed separately for ML and AP CoP displacement signals. Ellipse area (95% confidence ellipse) was derived from the covariance matrix of the two‐dimensional CoP trajectory [26]. Sample entropy (SampEn) was calculated independently for ML and AP CoP displacement signals using an embedding dimension of *m* = 2 and a tolerance of *r* = 0.2 × the signal standard deviation [27]. These parameters are commonly used in methodological studies of gait‐related CoP signals and were selected to facilitate comparison with previous work; however, SampEn estimates obtained from the 200‐point trajectories should be interpreted as specific to the selected data length and parameter settings [28, 29]. SampEn is typically interpreted as an index of temporal regularity or predictability, with higher values reflecting reduced regularity (i.e., greater unpredictability). Topological descriptors were extracted independently from each stance trial using Ripser. py (version 0.6.12) in Python. Each 200‐point ML–AP CoP trajectory was treated as a two‐dimensional point cloud. Vietoris–Rips persistence diagrams were computed using pairwise Euclidean distances with a maximum homology dimension of 1. No explicit removal of duplicate points, user‐defined maximum filtration threshold, or minimum persistence threshold was applied.

The infinite interval representing the final connected H0 component was excluded because it has no finite lifetime and therefore cannot be assigned a finite weight in the persistence‐entropy calculation. For each remaining finite positive‐persistence interval, persistence was defined as $l_{i}=d_{i}-b_{i}$, where $b_{i}\;$and $d_{i}$ denote its birth and death values, respectively. The interval lifetimes were normalized by their total persistence as $p_{i}=\frac{l_{i}}{\sum_{j=1}^{n}l_{j}}$. Persistence entropy was calculated separately for H0 and H1 as $\text{PE}=-\sum_{i=1}^{n}p_{i}\ln\left(p_{i}\right)$ [30]. No minimum persistence threshold was applied; therefore, all finite positive‐persistence intervals, including very short‐lived intervals, were retained. When no finite positive H1 interval was present, H1 persistence entropy was assigned a value of zero rather than treated as missing. Diagrams containing only one finite positive H1 interval also yielded zero persistence entropy because the normalized interval weight was equal to one. This procedure was applied consistently across all trials, and no trial was excluded solely because it contained no H1 feature. Trial‐level values were subsequently averaged within each participant, condition, and foot.

### 2.3 Statistical Analysis

Statistical analyzes were performed using mixed‐factor repeated‐measures ANOVA models. Group (PD vs. control) was treated as a between‐subject factor, whereas Task (NG vs. VRG), Foot (left vs. right), and, where applicable, Direction (AP vs. ML) were treated as within‐subject factors. Direction‐dependent variables (path length, velocity, RMS, and SampEn) were analyzed using four‐way mixed ANOVA models (Group × Task × Foot × Direction). Direction‐independent variables (ellipse area, H0 and H1 persistence entropy, and stance duration) were analyzed using three‐way mixed ANOVA models (Group × Task × Foot). Left and right feet were classified anatomically in both groups; the PD limbs were not categorized as more‐ and less‐affected. Trial‐level values were averaged within participant, condition, foot, and direction before inferential analysis. Assumptions of normality and homogeneity of variance were evaluated using Shapiro–Wilk and Levene's test, respectively. When sphericity assumptions were violated, Greenhouse–Geisser corrections were applied. Significant interactions were followed by simple‐effects comparisons based on estimated marginal means. For the significant Group × Task interaction, groups were compared separately within NG and VRG, with Bonferroni adjustment applied across the two comparisons. Simple‐effects analyzes were not conducted in the absence of a significant interaction. Effect sizes were reported as partial eta squared (ηp^2^). All statistical analyzes were performed using SPSS (Version 26, IBM Corp., Armonk, NY, USA), with the significance threshold set at α = 0.05.

## 3 Results

**Figure 1:**
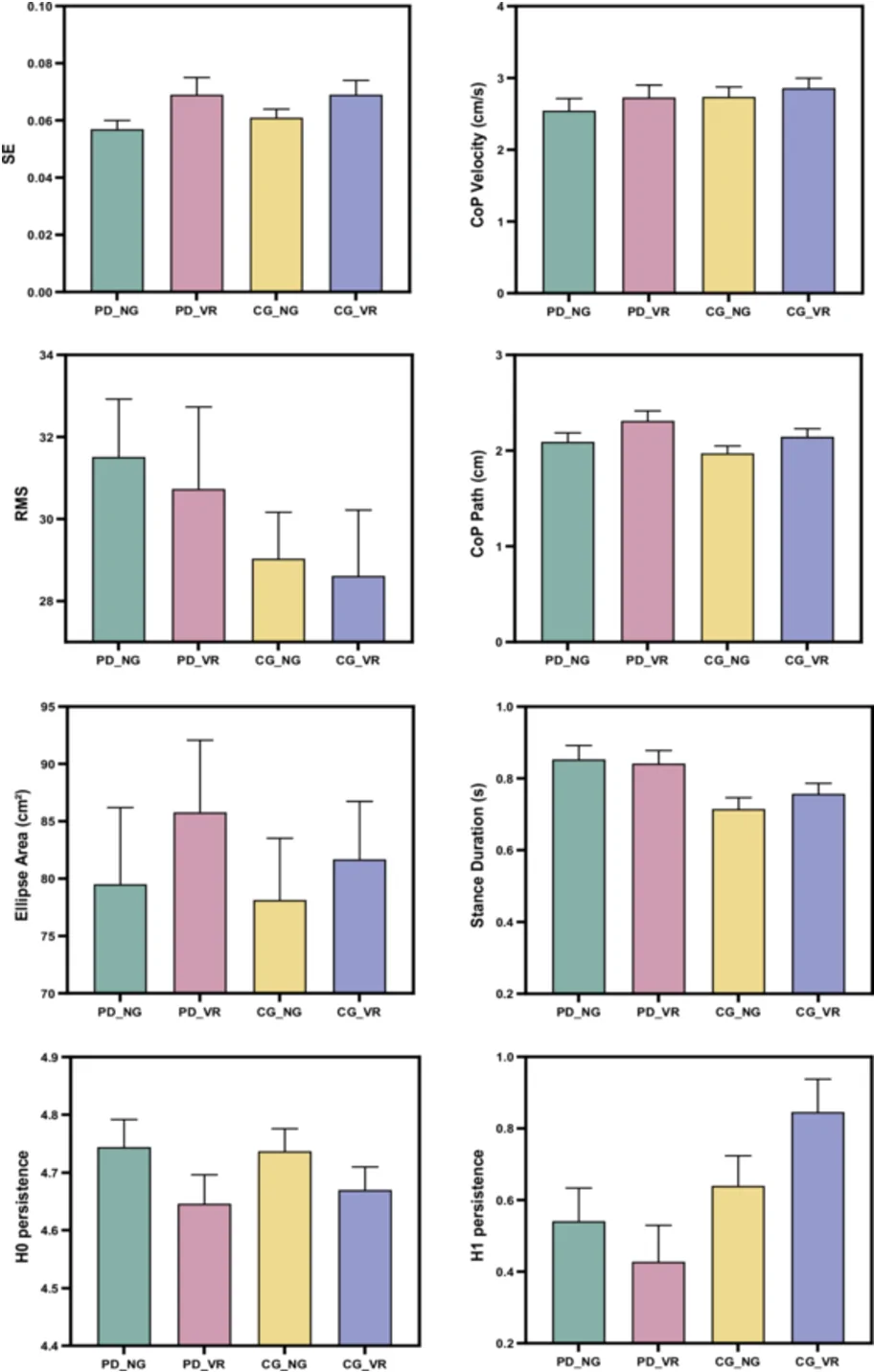
Group and task differences in stance‐phase center‐of‐pressure (CoP) dynamics during normal and virtual reality walking. Panels illustrate mean ± SEM values for sample entropy (SampEn), mean CoP velocity, root mean square (RMS) displacement, CoP path length, ellipse area, stance duration, H0 persistence entropy, and H1 persistence entropy in Parkinson's disease (PD) and control (CG) groups during normal gait (NG) and virtual reality (VR) walking.

Table 1 displays the demographic characteristics of the participants, as well as a comparison of these characteristics between the two groups. Table 2 summarizes the descriptive statistics and mixed‐factor ANOVA outcomes for all stance‐phase CoP variables, while Figure 1 illustrates the corresponding group‐ and task‐related patterns. Left‐ and right‐foot values were retained separately in the statistical models. No significant main effect of Foot or interaction involving Foot was observed for any outcome (all *p* > 0.05). Because the PD limbs were not classified as clinically more‐ and less‐affected, these null anatomical left‐versus‐right effects should not be interpreted as evidence for the absence of PD‐related asymmetry. Persistence diagrams containing no finite positive H1 interval were assigned an H1 persistence‐entropy value of zero and retained in the participant‐level averages; no trial was excluded solely because it lacked an H1 feature. Diagrams containing only one finite positive H1 interval also yielded zero persistence entropy.

**Table 1:** Demographic and clinical characteristics of participants in the Parkinson's disease (PD) and control groups.

	Groups		*p*‐value
	PD	CG	
*N* (female, male)	15 (7,8)	17 (8,9)	
Age (year)	61.60 ± 6.23	60.52 ± 6.17	0.62
Mass (Kg)	67.60 ± 10.56	68.88 ± 11.60	0.75
Height (m)	1.64 ± 0.10	1.64 ± 0.09	0.86
BMI (kg/m^**2**^ **)**	25.13 ± 3.39	25.71 ± 3.45	0.64

*Note:* Values are presented as mean ± standard deviation unless otherwise stated. *p*‐values reflect between‐group comparisons (independent samples *t*‐test).

Abbreviations: BMI, body mass index; CG, control group; PD, Parkinson's disease.

**Table 2:** Descriptive statistics and mixed‐factor ANOVA results for stance‐phase center‐of‐pressure (CoP) metrics during normal gait and virtual reality walking in Parkinson's disease and control groups.

		CG		PD		Group	Task	Group*Task	Direction	Group*Direction
		NG	VR	NG	VR					
SampEn	AP_Left	0.04 ± 0.01	0.05 ± 0.02	0.03 ± 0.01	0.03 ± 0.02	F = 0.098 *p* = 0.757 ηp^2^ = 0.004	F = 6.72 *p* = **0.015** ηp^2^= 0.21	F = 0.296 *p* = 0.591 ηp^2^ = 0.01	F = 120.16 *p* < **0.001** ηp^2^ = 0.82	F = 3.74 *p* = 0.064 ηp^2^ = 0.13
	AP_Right	0.04 ± 0.01	0.05 ± 0.02	0.04 ± 0.02	0.04 ± 0.03					
	ML_Left	0.09 ± 0.02	0.08 ± 0.03	0.08 ± 0.02	0.09 ± 0.04					
	ML_Right	0.07 ± 0.02	0.09 ± 0.03	0.07 ± 0.02	0.11 ± 0.04					
CoP Velocity (cm/s)	AP_Left	3.90 ± 0.81	4.10 ± 0.94	3.89 ± 1.24	3.70 ± 1.00	F = 0.643 *p* = 0.430 ηp^2^ = 0.02	F = 3.11 *p* = 0.089 ηp^2^ = 0.11	F = 0.134 *p* = 0.718 ηp^2^ = 0.005	F = 346.2 *p* < **0.001** ηp^2^ = 0.93	F = 0.098 *p* = 0.768 ηp^2^ = 0.003
	AP_Right	4.04 ± 0.70	4.02 ± 0.84	3.68 ± 1.18	3.88 ± 0.83					
	ML_Left	1.54 ± 0.39	1.70 ± 0.50	1.26 ± 0.45	1.65 ± 0.65					
	ML_Right	1.57 ± 0.42	1.62 ± 0.41	1.34 ± 0.36	1.68 ± 0.74					
RMS	AP_Left	45.36 ± 9.70	43.03 ± 9.32	50.82 ± 13.48	50.07 ± 12.30	F = 1.49 *p* = 0.234 ηp^2^ = 0.05	F = 0.264 *p* = 0.612 ηp^2^ = 0.01	F = 0.024 *p* = 0.878 ηp^2^ = 0.001	F = 191.59 *p* < **0.001** ηp^2^ = 0.88	F = 1.36 *p* = 0.254 ηp^2^ = 0.05
	AP_Right	45.23 ± 7.74	45.80 ± 22.83	50.27 ± 13.25	49.25 ± 14.10					
	ML_Left	12.29 ± 3.05	12.61 ± 3.60	11.26 ± 4.42	10.69 ± 3.47					
	ML_Right	13.25 ± 4.21	13.01 ± 3.32	13.69 ± 3.91	12.91 ± 6.04					
CoP Path (cm)	AP_Left	2.78 ± 0.51	3.09 ± 0.59	3.16 ± 0.91	3.12 ± 0.61	F = 1.62 *p* = 0.214 ηp^2^ = 0.06	F = 9.64 *p* = **0.005** ηp^2^ = 0.27	F = 0.146 *p* = 0.705 ηp^2^ = 0.006	F = 463.01 *p* < **0.001** ηp^2^ = 0.95	F = 1.39 *p* = 0.249 ηp^2^ = 0.05
	AP_Right	2.87 ± 0.39	2.99 ± 0.52	3.03 ± 0.55	3.40 ± 0.86					
	ML_Left	1.11 ± 0.25	1.29 ± 0.40	1.05 ± 0.25	1.31 ± 0.40					
	ML_Right	1.12 ± 0.28	1.21 ± 0.24	1.12 ± 0.20	1.42 ± 0.45					
Ellipse Area (mm^2^)	Left	68.06 ± 17.45	80.19 ± 33.42	72.14 ± 21.67	74.85 ± 19.30	F = 0.157 *p* = 0.695 ηp^2^ = 0.006	F = 1.09 *p* = 0.305 ηp^2^ = 0.04	F = 0.085 *p* = 0.772 ηp^2^ = 0.003		
	Right	88.22 ± 28.43	83.17 ± 26.59	86.87 ± 33.93	96.72 ± 32.77					
H0 persistence entropy	Left	4.75 ± 0.19	4.63 ± 0.17	4.73 ± 0.26	4.65 ± 0.25	F = 0.022 *p* = 0.884 ηp^2^ = 0.001	F = 11.68 *p* = **0.002** ηp^2^ = 0.31	F = 0.436 *p* = 0.515 ηp^2^ = 0.016		
	Right	4.73 ± 0.22	4.71 ± 0.16	4.75 ± 0.20	4.64 ± 0.16					
H1 persistence entropy	Left	0.68 ± 0.35	0.84 ± 0.39	0.57 ± 0.32	0.40 ± 0.35	F = 5.22 *p* = **0.030** ηp^2^ = 0.15	F = 1.44 *p* = 0.241 ηp^2^ = 0.05	F = 5.69 *p* = **0.025** ηp^2^ = 0.16		
	Right	0.60 ± 0.50	0.85 ± 0.37	0.51 ± 0.42	0.45 ± 0.38					
Stance Duration (s)	Left	0.71 ± 0.08	0.75 ± 0.11	0.86 ± 0.19	0.82 ± 0.13	F = 6.91 *p* = **0.014** ηp^2^ = 0.21	F = 0.477 *p* = 0.496 ηp^2^ = 0.02	F = 1.42 *p* = 0.244 ηp^2^ = 0.05		
	Right	0.71 ± 0.07	0.76 ± 0.12	0.85 ± 0.18	0.86 ± 0.13					

*Note:* Values are presented as mean ± standard deviation (SD). Mixed‐factor repeated‐measures ANOVA examined the effects of Group (Parkinson's disease [PD] vs. control group [CG]), Task (normal gait [NG] vs. virtual reality walking [VR]), and Direction (anteroposterior [AP] vs. mediolateral [ML], where applicable). For direction‐dependent variables (SampEn, CoP velocity, RMS, and CoP path length), Group × Task × Direction models were applied. For direction‐independent variables (ellipse area, H0 persistence entropy, H1 persistence entropy, and stance duration), Group × Task models were used. F values, *p* values, and partial eta squared (ηp^2^) effect sizes are reported.

Abbreviations: CoP, center of pressure; RMS, root mean square; SampEn, sample entropy; H0/H1, homology dimensions 0 and 1; SD, standard deviation; ηp^2^, partial eta squared.

### 3.1 Linear CoP Magnitude Measures

Across velocity, RMS, path length, and ellipse area, no significant main effect of Group was observed (all *p* > 0.05), and no Group × Task interaction reached significance. A significant main effect of Task was detected only for CoP path length (F(1, 30) = 9.64, *p* = 0.005, ηp^2^ = 0.27), indicating greater CoP excursion during VR than during normal gait across groups. Task effects were not significant for velocity (*p* = 0.089), RMS (*p* = 0.612), or ellipse area (*p* = 0.305), although descriptive values were higher under VR. A robust main effect of Direction was consistently observed for velocity (ηp^2^ = 0.93), RMS (ηp^2^ = 0.88), and path length (ηp^2^ = 0.95), with larger magnitudes in the AP direction compared to ML. No significant Group × Direction interactions were found.

### 3.2 Nonlinear Complexity (Sample Entropy)

Sample entropy showed a significant main effect of Task (F(1, 30) = 6.72, *p* = 0.015, ηp^2^ = 0.21), reflecting an overall increase of approximately 22% during VR gait. Neither the main effect of Group nor the Group × Task interaction was significant, indicating no evidence that the task‐related change differed between groups. A strong main effect of Direction was observed (F(1, 30) = 120.16, *p* < 0.001, ηp^2^ = 0.82), with higher SampEn in the ML direction.

### 3.3 Persistence Entropy and Stance Duration

H0 persistence entropy showed a significant main effect of Task (F(1, 30) = 11.68, *p* = 0.002, ηp^2^ = 0.31), with lower values during VR across groups. Neither the Group effect nor the Group × Task interaction was significant. H1 persistence entropy showed significant Group (F(1, 30) = 5.22, *p* = 0.030, ηp^2^ = 0.15) and Group × Task effects (F(1, 30) = 5.69, *p* = 0.025, ηp^2^ = 0.16). Bonferroni‐adjusted comparisons of estimated marginal means indicated a significant between‐group difference during VR (F(1, 30) = 9.23, *p* = 0.005, ηp^2^ = 0.24), but not during normal gait (F(1, 30) = 0.64, *p* = 0.431, ηp^2^ = 0.02). Descriptively, H1 persistence entropy increased from normal gait to VR in controls and decreased in PD. Stance duration showed a significant main effect of Group (F(1, 30) = 6.91, *p* = 0.014, ηp^2^ = 0.21), with longer stance duration in PD across tasks. Neither the Task effect nor the Group × Task interaction was significant.

Among the 353 available trial‐level observations from the normal‐gait and VR tasks, 83 (23.5%) contained no finite H1 features, 30 (8.5%) contained exactly one H1 feature, and 240 (68.0%) contained two or more H1 features. In the control group, zero‐, one‐, and multiple‐feature diagrams accounted for 21 (21.4%), 11 (11.2%), and 66 (67.3%) of the 98 normal‐gait observations, respectively, and 12 (12.5%), 5 (5.2%), and 79 (82.3%) of the 96 VR observations, respectively. In the PD group, the corresponding frequencies were 27 (31.4%), 9 (10.5%), and 50 (58.1%) among 86 normal‐gait observations and 23 (31.5%), 5 (6.8%), and 45 (61.6%) among 73 VR observations. H1 persistence entropy was zero for both zero‐feature and one‐feature diagrams, and these observations were retained according to the predefined zero‐assignment rule.

## 4 Discussion

This study investigated whether walking in a VR environment alters CoP dynamics and their topological organization during stance in individuals with PD and healthy older adults. VR increased CoP path length across groups, whereas other linear magnitude measures, including velocity, RMS, and ellipse area, showed no significant Task or Group effects. SampEn increased during VR overall, without evidence that the task‐related change differed between groups. H0 persistence entropy decreased in both groups during VR. In contrast, H1 persistence entropy showed significant Group and Group × Task effects, with descriptive increases during VR in controls and decreases in PD. Stance duration was longer in PD regardless of task. Overall, walking in the altered VR context was associated with changes that were only partly captured by conventional CoP magnitude measures. A group‐dependent response was observed for H1 persistence entropy across the two walking conditions.

For linear CoP magnitude measures, the primary task effect was an overall VR‐related increase in CoP path length across groups, with consistently greater values in the AP than ML direction. In biomechanical terms, increased path length under more demanding conditions is typically interpreted as greater CoP excursion, potentially reflecting enhanced corrective activity or altered neuromuscular coordination [31]. VR has been shown to modify walking behavior in PD, particularly when perceptual constraints or visuospatial demands are imposed [1], and can influence spatiotemporal parameters and freezing‐related outcomes [3]. Thus, the increased CoP path length observed during VR is consistent with VR‐induced adjustments in locomotor control. Despite longer stance duration in PD, no significant group effects or Group × Task interactions were found for velocity, RMS, path length, or ellipse area. This is consistent with evidence suggesting that kinetic‐based dynamic markers may be less sensitive than kinematic variability measures in heterogeneous PD cohorts [32, 33]. However, other studies report that CoP‐derived features can detect PD‐related abnormalities, including stance‐phase asymmetries [8]. Methodological context likely contributes to these discrepancies: treadmill and instrumented‐walkway paradigms can produce CoP patterns that differ from overground walking, even in healthy adults [34]. Although left‐ and right‐foot values were retained separately and Foot was included in the statistical models, no Foot‐related effect was significant. However, anatomical left–right comparisons do not necessarily correspond to the more‐ and less‐affected sides in PD. Therefore, clinically meaningful disease‐related asymmetry may not have been captured by the present analysis [8].

SampEn increased by approximately 22% during VR gait across groups. SampEn is commonly used as an index of signal regularity or predictability, with higher values indicating less predictable fluctuations [28, 35]. Importantly, greater entropy does not necessarily reflect healthy complexity; single‐scale entropy metrics primarily quantify regularity rather than multiscale physiological complexity [9]. Accordingly, the VR‐related increase should be interpreted as reduced CoP predictability rather than clear improvement or impairment. Previous research indicates that CoP SampEn can be sensitive to changes in task and environmental context [28, 36]. However, because cognitive load, attentional demand, sensory conflict, optic‐flow processing, cybersickness, and perceived task difficulty were not measured, the mechanism underlying this change cannot be determined. The strong Direction effect, characterized by higher SampEn in ML than AP, may reflect the distinct control requirements of mediolateral balance regulation [37, 38]. However, the nonsignificant Group and Group × Task effects provide no evidence of different SampEn levels or VR‐related responses between the PD and control groups.

The most distinctive group‐specific finding concerned H1 persistence entropy. H0 persistence entropy decreased during VR across groups, indicating a less even distribution of finite H0 lifetimes. H1 persistence entropy showed a significant Group × Task interaction, with descriptive increases during VR in controls and decreases in PD. However, this finding cannot be attributed solely to PD diagnosis because relevant clinical heterogeneity was not incorporated into the analysis. Persistence entropy describes the relative distribution of finite lifetimes rather than their number or absolute magnitude [11, 30]; therefore, the interaction does not establish stronger or more persistent loop‐like features. An important interpretative limitation is that H1 persistence entropy was assigned a value of zero when no finite H1 feature was present and was also mathematically equal to zero when exactly one feature was present. These topologically distinct situations were therefore numerically indistinguishable in the entropy measure. Moreover, because the attainable range of persistence entropy depends partly on the number of finite features, the observed Group × Task interaction cannot be attributed exclusively to differences in the relative distribution of H1 persistence lifetimes. Differences in the occurrence or number of H1 features may also have contributed to the finding. Because zero‐ and one‐feature diagrams were retained, the group‐level H1 persistence‐entropy estimates reflect a combination of feature occurrence, feature number, and the relative distribution of feature lifetimes. Accordingly, the present H1 finding should be regarded as exploratory and not as an isolated measure of persistence‐lifetime organization.

Direct evidence linking persistent homology of CoP trajectories to specific neural mechanisms remains limited; however, recent studies support the utility of topological descriptors as sensitive markers of gait‐ and pathology‐related structure. Topology‐based gait analyzes have demonstrated that TDA‐derived features capture clinically relevant information beyond conventional spatiotemporal metrics [39]. Persistent homology has been applied to characterize PD‐related gait alterations and to distinguish PD from controls based on movement trajectories [15], and persistent entropy has been incorporated into PD severity classification frameworks, reflecting growing translational interest [14]. Collectively, this literature supports the exploratory use of topological descriptors in gait analysis. In the primary analysis, H1 persistence entropy showed a group‐dependent pattern across walking conditions; however, because perceptual, cognitive, sensory, and neurophysiological variables were not measured and sensitivity analyzes across alternative point‐cloud densities and persistence thresholds were not performed, its underlying mechanism and robustness to alternative analytical settings cannot be determined [40].

Stance duration was longer in the PD group, with no significant effect of task. Prolonged stance time, often accompanied by increased double‐support duration, is consistent with established characterizations of parkinsonian gait as slower and more cautious, with altered temporal organization [41]. The absence of a task effect is notable, as VR has been shown to modify spatiotemporal gait parameters in PD. Because potential perceptual and cognitive mechanisms were not measured, the absence of a Task effect on stance duration cannot be attributed to a specific control process.

Several limitations should be acknowledged. First, the modest and clinically heterogeneous PD sample limited subgroup analyzes, and relevant factors—including disease severity and duration, medication dose, freezing and fall history, motor phenotype, affected side, and cognitive performance—were unavailable or not incorporated into the analysis. Thus, the H1 persistence‐entropy response cannot be attributed solely to PD diagnosis, and disease‐related asymmetry could not be directly evaluated. Second, the limited number of valid trials and the dependence of SampEn and persistent homology on data length, parameter selection, and preprocessing may affect the robustness of the findings [29]. In particular, all CoP trajectories were resampled to 200 points as a pragmatic standardization choice rather than an empirically optimized point‐cloud density, and no minimum persistence threshold was applied. Consequently, the number and lifetime distribution of H1 features may have been influenced by interpolation density and the inclusion of very short‐lived features. Because sensitivity analyzes across alternative resampling densities or minimum‐persistence thresholds were not performed, it remains unknown whether the observed Group × Task interaction for H1 persistence entropy would remain stable under alternative analytical settings. The primary H1 finding should therefore be regarded as exploratory and requires replication using prespecified resampling densities and persistence‐threshold criteria. Finally, because VR systems differ in immersion and visual characteristics, the findings may not generalize to other VR configurations.

## 5 Conclusion

Walking within a VR environment increased CoP excursion and reduced CoP predictability, while also altering the topological organization of stance‐phase CoP trajectories. H1 persistence entropy showed a group‐dependent response across normal and VR walking; however, because zero entropy represented both zero‐ and one‐feature diagrams and the attainable entropy depends partly on feature count, this response may reflect differences in the occurrence or number of finite H1 features as well as in the relative distribution of their lifetimes. H1 persistence entropy should therefore be considered an exploratory topological descriptor rather than a direct measure of gait‐control adaptability. Its physiological interpretation and clinical relevance require further validation.

## Author Contributions

Elaheh Azadian and Mahdi Majlesi contributed substantially to the study conception and design, data analysis and interpretation, and article drafting. Alireza Ardalany Moghadam was responsible for data extraction, preprocessing, and methodological support. Rezvan Bakhtiarian conducted data collection and experimental testing. Makwan Jabar Ali contributed to critical revision of the article for important intellectual content and interpretation of findings. All authors reviewed the article, provided feedback, and approved the final version for publication.

## Funding

The authors have nothing to report.

## Ethics Statement

The study protocol was approved by the Ethics Committee of Islamic Azad University, Hamedan Branch (approval code: IR.IAU.H.REC.1401.001). All procedures were conducted in accordance with the Declaration of Helsinki, and written informed consent was obtained from each participant prior to enrollment.

## Consent

All authors have consented to the publication of this article.

## Conflicts of Interest

The authors declare no conflicts of interest.

## Transparency Statement

The corresponding author, Elaheh Azadian, affirms that this manuscript is an honest, accurate, and transparent account of the study being reported; that no important aspects of the study have been omitted; and that any discrepancies from the study as planned have been explained.

## Data Availability

The data that support the findings of this study are available from the corresponding author upon reasonable request.
